# Crizotinib: aseptic abscesses in multiple organs during treatment of EML4-ALK-positive NSCLC

**DOI:** 10.1007/s00432-021-03664-w

**Published:** 2021-08-09

**Authors:** Daniel Weber, Miriam Decker, Michael Schuster, Sara Folz, Carsten Johannes Stürmer, Manfred P. Lutz

**Affiliations:** 1 Department of Internal Medicine, Caritasklinikum St. Theresia, Rheinstrasse2, 66113 Saarbrücken, Germany; 2 Centre Hospitalier de Luxembourg, Luxembourg, Luxembourg

**Keywords:** Crizotinib, EML4-ALK, NSCLC, Aseptic abscesses, Side effect

## Abstract

**Purpose:**

We report a novel side effect of Crizotinib, an oral ALK inhibitor used in the treatment of non-small cell lung cancer (NSCLC) with activating rearrangement of EML4-ALK. It expands the known spectrum of complications of Crizotinib.

**Methods:**

Clinical case report.

**Results:**

Multiple aseptic and recurrent abscesses were observed in the liver, thoracic wall as well as in both kidneys in a 75-year-old female patient suffering from NSCLC who had been treated with Crizotinib for almost 2 years. After discontinuation of the treatment the abscesses dissolved spontaneously and did not reoccur.

**Conclusion:**

Aseptic abscesses under treatment with Crizotinib are not restricted to the kidneys as described before, but can also occur in other abdominal organs as the liver and even in the thoracic wall. We postulate that this finding may point to a yet unknown not tissue-dependent mechanism of action.

Crizotinib is a targeted first-generation oral inhibitor of the anaplastic lymphoma kinase (ALK). It is commonly used for the treatment of non-small cell lung cancers (NSCLC) with activating rearrangements of EML4-ALK (Kwak et al. 2010; Soda et al. 2007). Treating advanced ALK-positive NSCLC-patients with Crizotinib improves progression-free survival, reduces symptoms and improves global quality of life when compared to chemotherapy (Shaw et al. 2013).

Common adverse events under treatment with crizotinib include malaise, nausea, vomiting, visual problems, elevated transaminases, and interstitial lung disease. In addition, several studies described the occurrence of renal cysts in up to 4% of the patients, some of them even affecting neighbouring structures (Yasuma et al. 2018).

Here, we report the case of a 75-year-old non-smoking woman who developed multiple aseptic hepatic, thoracic and renal abscesses under treatment with crizotinib. The patient presented initially in 2016 with an advanced adenocarcinoma staged cT4 cN2 cM1a (contralateral lung segment 10), harbouring an EML4-ALK rearrangement. After two courses of chemotherapy, second line therapy with crizotinib was started leading to complete remission within 6 months. Ten months after the start of crizotinib, a tumorous formation on the right kidney was detected. Surgical resection showed an aseptic abscess. One year later, the patient presented with fatigue, inappetence, intermittent sweating and diffuse abdominal pain. Computed tomography revealed two large abscesses with typical rim enhancement in the left-sided thoracic wall (measuring 7.2 × 3.6 × 1.9 cm), left liver lobe (9.6 × 7.7 × 4.5 cm) and several others in the left kidney, right liver lobe and the interenteric space (Figs. 1, 2).

**Fig. 1:**
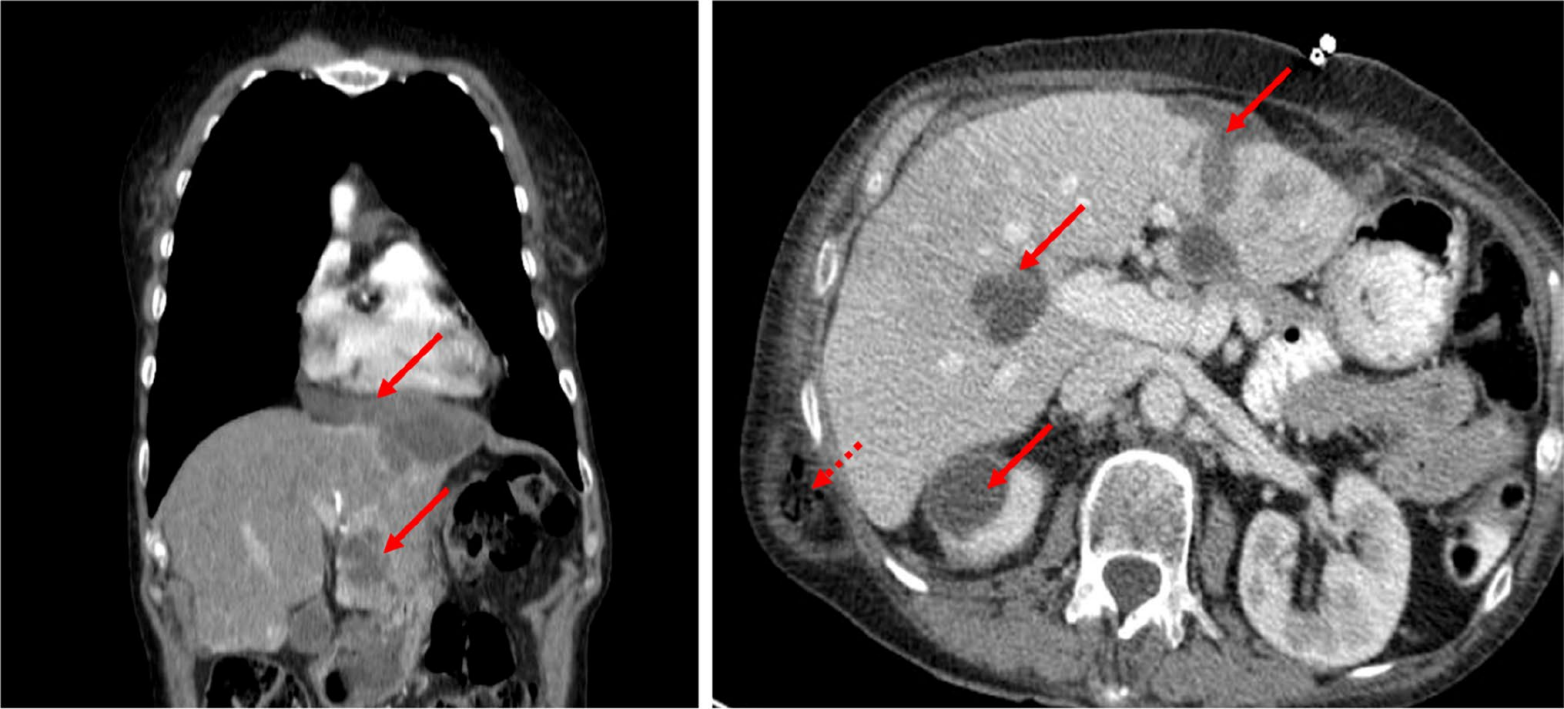
Computed tomography scan after 2 years of treatment with crizotinib showing abscess formations (arrows) in the liver, the thoracic wall and kidney. The dashed arrow indicates a subcutaneous hernia

**Fig. 2:**
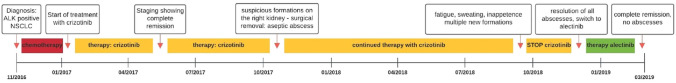
Timeline of key events:

Laboratory evaluation demonstrated normal WBC count, elevated CRP (140 mg/l) and slightly elevated PCT (0.8 ng/ml) as well as increased liver function tests (below twice the ULN). Repeated blood cultures were negative. Under the suspicion of an infection, percutaneous ultrasound-guided drainage of the abscesses at the left kidney and the liver was performed. It yielded blood-stained serous fluid, cytologically described as abscess without tumour cells. Microbiological culture as well as screening for extrapulmonary tuberculosis or atypical mycobateriosis including Ziehl–Neelsen staining and PCR was negative. Endoscopic examination of the colon as well as transthoracic and transesophageal echocardiography did not find any other source of infection. Intravenous treatment with piperacillin/tazobactam was without effect. Therefore, Crizotinib was discontinued, which led to rapid improvement of the patients overall condition and symptoms. CRP and the impaired liver function tests normalised and sonographic follow-up showed ongoing global resolution of all abscesses, including those without drainage. Treatment was changed to Alectinib, with maintained complete remission without cyst formation.

In summary we describe for the first time the occurrence of multiple aseptic abscesses in various tissue types during treatment with Crizotinib. Even though renal cyst formation is a well-known complication, the postulated modulation of the c-MET and ROS1 tyrosine kinase in renal tubular epithelium does not explain our observation. We hypothesise that off-target inhibition of yet another ubiquitously expressed target may be responsible. One possible candidate could be the focal adhesion kinase, which regulates cyst formation through modulation of the focal adhesion complex and is a known target of crizotinib (Israeli et al. 2010; Troutman et al. 2016).

## Data Availability

Informed consent.
